# Role of pH-induced structural change in protein aggregation in foam fractionation of bovine serum albumin

**DOI:** 10.1016/j.btre.2016.01.002

**Published:** 2016-01-22

**Authors:** Rui Li, Zhaoliang Wu, Yanji Wangb, Linlin Ding, Yanyan Wang

**Affiliations:** a School of Chemical Engineering and Technology, Hebei University of Technology, No.8 Guangrong Road, Dingzi Gu, Hongqiao District, Tianjin, 300130, China; b Key Laboratory of Green Chemical Technology & High Efficient Energy Saving, Hebei University of Technology, No. 8 Guangrong Road, Dingzi Gu, Hongqiao District, Tianjin, 300130, China; c Lianyungang TCM Branch of Jiangsu Union Technical Institute, Jiangsu Lianyungang 222006, China

**Keywords:** Protein aggregation, Foam fractionation, pH, Molecular structure, BSA

## Abstract

For reducing protein aggregation in foam fractionation, the role of pH-induced structural change in the interface-induced protein aggregation was analyzed using bovine serum albumin (BSA) as a model protein. The results show that the decrease in pH from 7.0 to 3.0 gradually unfolded the BSA structure to increase the molecular size and the relative content of β-sheet and thus reduced the stability of BSA in the aqueous solution. At the isoelectric point (pH 4.7), BSA suffered the lowest level in protein aggregation induced by the gas–liquid interface. In the pH range from 7.0 to 4.7, most BSA aggregates were formed in the defoaming process while in the pH range from 4.7 to 3.0, the BSA aggregates were formed at the gas–liquid interface due to the unfolded BSA structure and they further aggregated to form insoluble ones in the desorption process.

## Highlights

• pH effects on foam-induced BSA aggregation were studied at the molecular level.
• Structural unfolding more readily induced BSA aggregation than zero net charge.
• pH determined the process in which BSA aggregation occurred.
• BSA suffered the lowest level in aggregation at its isoelectric point.

## Graphical abstract

**Figure:**
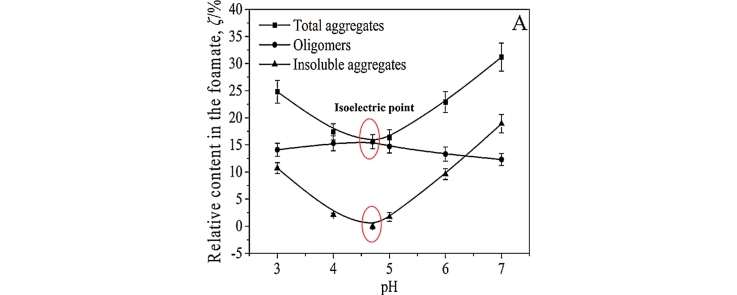

## 1 Introduction

Foam fractionation is a physical process in which rising foam serves as the medium to separate surface-active compounds from their diluted solutions [24]. Due to its low cost, free pollution and high efficiency at low concentrations, foam fractionation is widely used to separate not only various organic and inorganic chemical compounds, but also biological materials such as proteins and microorganisms [20], [5], [6]. A typical example for its industrial application is that it is successfully used by Tianjin Kangyi Biotechnology Company in China to separate nisin (a polypeptide) from the fermentation broth. By replacing membrane separation, foam fractionation has effectively decreased the price of nisin from 1200 RMB/kg at 2004 to 500 RMB at 2006 and then to 300 RMB/kg at 2015 (the data were obtained from Tianjin Kangyi Biotechnology Company). The successful application gives foam fractionation a great potential in the large-scale separation of proteins from their highly diluted aqueous solutions [6].

**Fig. 1:**
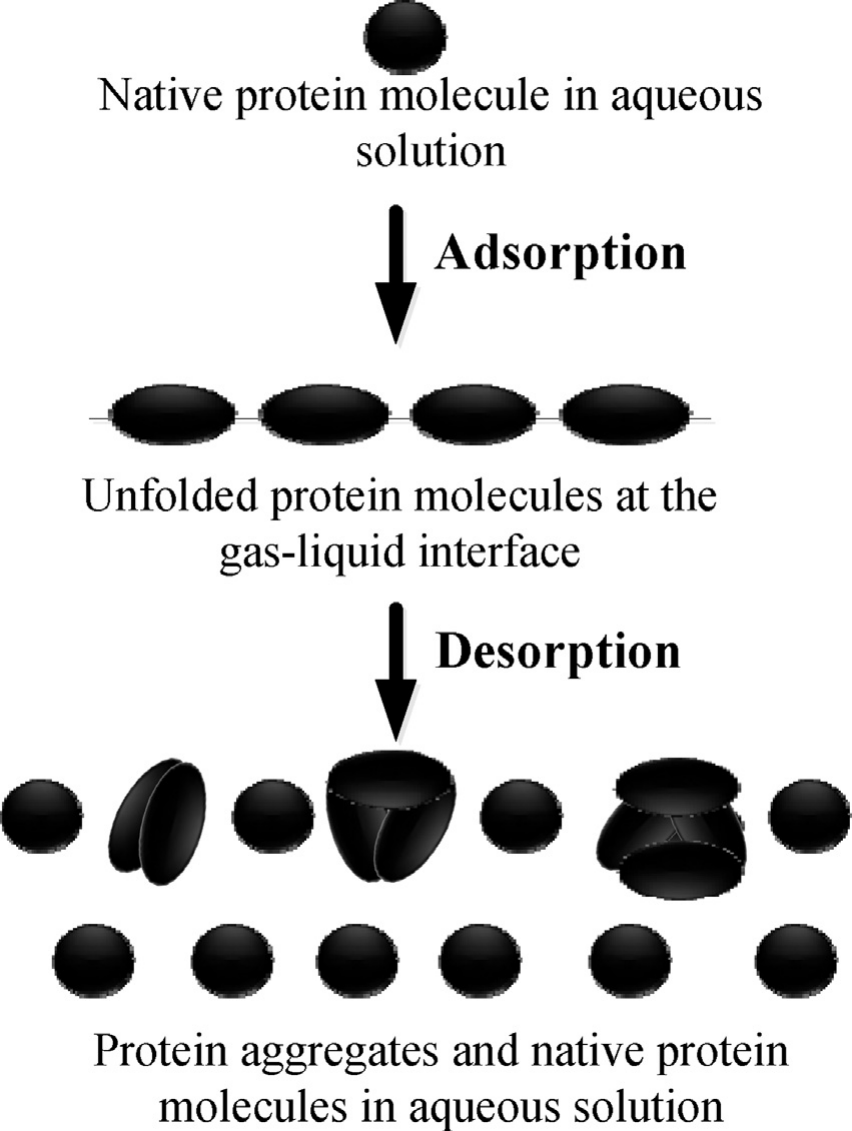
Schematic diagram of protein aggregation induced by the gas–liquid interface.

Foam fractionation has been troubled by protein aggregation induced by the gas–liquid interface [7]. For clearly describing how protein aggregation was induced by the gas–liquid interface, a schematic diagram is presented in Fig. 1. It is presented that protein molecules suffer structural unfolding as they are adsorbed at the gas–liquid interface [1]. Because a large gas–liquid interfacial area is used in foam fractionation, the number of protein molecules that suffer denaturation at the interface will be large. In the desorption of the unfolded molecules from the interface, some of them cannot return their native structures so that they readily interact with each other to form aggregates [3], as presented in Fig. 1. In the foam fractionation of proteins, foam drainage is often enhanced to obtain a high enrichment ratio. However, the enhanced foam drainage reduces the gas–liquid interfacial area to intensify the desorption of adsorbed protein molecules in the rising foam. In this case, protein aggregates will flow into the bulk solution with the drained liquid and may reduce the protein recovery. Furthermore, the enhanced foam drainage increases the relative content of the adsorbed protein molecules in the foam so that protein aggregation in the defoaming process will also be intensified [17]. As a result, protein aggregation induced by the gas–liquid interface will significantly decrease the foam fractionation performances [18], [19]. Then it is necessary to give a molecular-level understanding of the gas–liquid interface-induced protein aggregation to reduce its bad effect on foam fractionation of proteins.

pH is a common parameter that significantly affects both protein aggregation and foam fractionation performances due to its effects on the zeta potentials and structures of proteins [29], [15]. So it is necessary to study the role of pH in protein aggregation in foam fractionation at the molecular level. In general, the net charge of a protein increases as pH deviates from its isoelectric point. The increased charge enhances the electrostatic repulsion between protein molecules to reduce their aggregation in the aqueous solution and reduce the protein surface excess at the gas–liquid interface [10], [13]. The decreased surface excess will result in an unstable foam with large bubbles and low volumetric liquid fraction. Then, a high protein enrichment ratio will be obtained so that a high-level protein aggregation in the defoaming process will be caused [17]. Furthermore, a pH far away from the protein isoelectric point also can unfold the protein structure to enhance protein aggregation [9]. Thus pH will have a complicated effect on the protein aggregation in foam fractionation.

Bovine serum albumin (BSA) is a typical globular protein which has a similar structure to human serum albumin (HSA), so it has wide applications in the pharmaceutical field [26], [16]. Due to its low price, high purity and good foamability, BSA is also widely used in the fundamental studies of foam fractionation [14]. Thus in this work, it will be selected as a model protein for the molecular-level study of the role of pH in protein aggregation in foam fractionation. At present, there are many references reporting the effects of pH on the structure and the foam fractionation of BSA [25], [30], [2]. However, few of them have reported the effect of pH on the aggregation of BSA in foam fractionation.

In the current work, the effects of pH on the zeta potential, secondary structure, tertiary structure and molecular size of BSA will be firstly investigated in the pH range from 7.0 to 3.0. Then, the effect of pH on the aggregation of BSA in the aqueous solution will be investigated. Subsequently, the effects of pH on the BSA surface excess and bubble size will be studied. Finally, the effects of pH on the relative contents of the BSA aggregates in the foamate will be investigated. Based on the above results, the role of pH in the aggregation of BSA in foam fractionation will be discussed at the molecular level.

## 2 Materials and methods

### 2.1 Materials

BSA (purity >99%) was purchased from Tianjin Unite Stars Biotechnology Co., Ltd., China. It was dissolved in ultrapure water (electrical resistance = 18.25 MΩ) prepared by UPR-II-10T water purification system (Chengdu Ultrapure Technology Co., Ltd., China). The pH of each BSA solution was adjusted by sodium hydroxide (NaOH, AR) and hydrochloric acid (HCl, w/w ≥ 37.0%) purchased from Tianjin Yingdaxigui Co., Ltd., China. The pH values of the BSA solutions were 7.0, 6.0, 5.0, 4.7, 4.0 and 3.0. 1,8-anilinonaphthalenesulfonate (ANS, HPLC purity ≥97%) was purchased from Sigma–Aldrich, USA.

### 2.2 Equipment

**Fig. 2:**
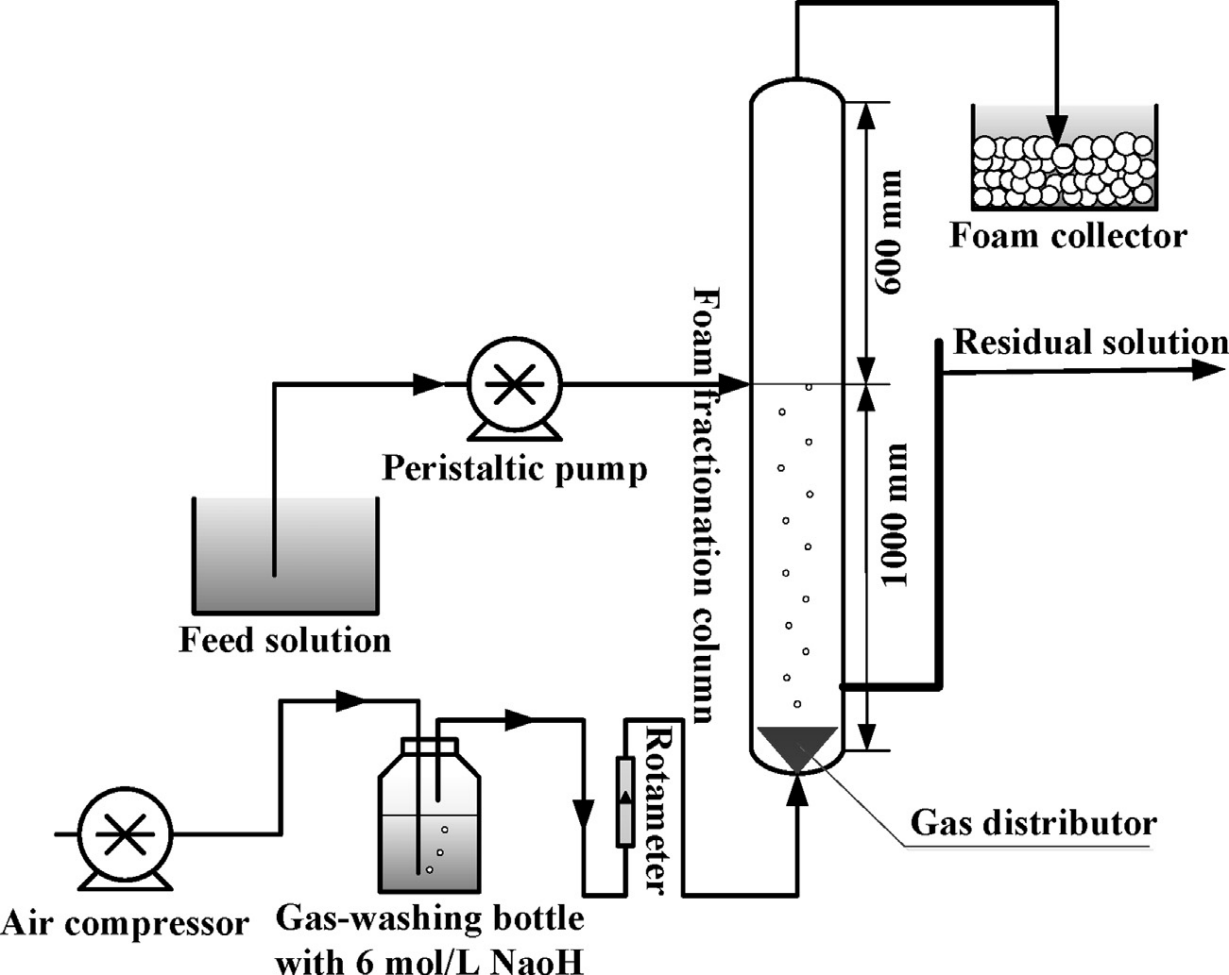
Schematic diagram of continuous foam fractionation of BSA.

Fig. 2 shows the equipment for the continuous foam fractionation of BSA. The foam fractionation column was constructed by a transparent perspex tube of 50 mm in inner diameter and 1600 mm in height. The BSA solution of 0.30 g/L was injected into the foam fractionation column by a peristaltic pump (BT04F, Beijing Yidaxinkang Precision Pump Co., Ltd., China). Its inlet was located at the foam–liquid interface which has a distance of 1000 mm from the column bottom. A gas distributor of lacunaris sintered glass with 100 ± 20 μm in pore diameter was mounted at the bottom of the column and it had a diameter of 25 mm. Through the distributor, the air was bubbled into the foam fractionation column by an air compressor (AC0-318, Guangdong Hailea Group Co., Ltd., China). In addition, CO_2_ in the air was removed by 6 mol/L NaOH solution to prevent its interference with the solution pH. In the experiments, feed flow rate, air flow rate and temperature were fixed at 20 mL/min, 100 mL/min and 25 ± 1 °C, respectively. All the collected foams were freely collapsed.

### 2.3 Measurements of zeta potential and molecular size distribution of BSA

Zeta potential and molecular size distribution of each BSA solution were measured with a Zetasizer Nano ZS90 (Malvern Instruments, England) at 25.0 ± 0.5 °C. Before each measurement, the sample solution was filtered with a 0.22 μm cellulose acetate filter and a thoroughly acid cleaned glass syringe. The BSA concentrations for measuring zeta potential and molecular size distribution were 15.0 g/L and 0.30 g/L, respectively.

### 2.4 Measurement of far-UV CD spectra of BSA solution

The secondary structures of BSA were measured on the basis of the far-UV CD spectra measured by using a Jasco J-810 spectral polarimeter (JASCO, Japan) with a water jacketed glass cuvette of 0.1 mm in optical path length at 25.0 ± 0.5 °C. The CD spectra of each BSA solution at 0.30 g/L in BSA concentration and its reference solution were collected in the range from 190 nm to 240 nm, with a resolution of 1 nm, a time response of 0.5 s and a screening speed of 100 nm/min. Each spectrum was an average of 8 accumulations. After the sample and the reference spectra were obtained, baseline correction was carried out using the spectra analysis software from Jasco. The secondary structure of BSA was estimated using Dichro Web Online K2D algorithms with a mean residual weight (MRW) of 114.1.

### 2.5 Measurement of extrinsic fluorescence spectrum of BSA solution

The extrinsic fluorescence spectra of BSA solutions were measured using an F-4500 fluorescence spectrophotometer (Hitachi Co., Japan) in the presence of the fluorescence probe ANS at 25.0 ± 0.5 °C. The addition of ANS solution (8.0 mmol/L) to each BSA solution was 1/200 (*v*/*v*). The spectrum of each BSA solution was collected at a scanning speed of 240 nm/min and an excitation wavelength of 370 nm with slits of 2.5 nm and 5.0 nm for excitation and emission, respectively. The spectra were in the range from 400 nm to 650 nm and each one was an average of 8 accumulations.

### 2.6 Measurement of surface tension of BSA solution

The surface tension of each BSA solution at 0.30 g/L was measured by the du Noüy ring method using a JYW-200C automatic surface tensiometer (Chengde Dingshen Testing Equipment Co., Ltd., China) at 25.0 ± 0.5 °C. The surface tension was measured after each solution stood for 20 min to obtain the adsorption equilibrium of BSA at the liquid surface.

### 2.7 Evaluation of enrichment and aggregation of BSA in foam fractionation

The enrichment and aggregation of BSA in foam fractionation were evaluated by enrichment ratio (*E*) and relative contents of BSA aggregates in the foamate (ζ), respectively. The former was defined as Eq. (1) while the latter and all the BSA concentrations were determined as the previous work of Li et al. [17] with a flow rate of mobile phase of 0.7 mL/min.

(1) $$E=\frac{C_{\text{f}}}{C_{\text{o}}}$$

where *C*_f_ and *C*_o_ are the BSA concentrations in the foamate and the feed BSA solutions, respectively, g/L.

### 2.8 Statistical analysis

Each of the experiments was at least triply repeated. An analysis of variance of the data was performed by using Microsoft Excel. The *t*-test with *p* ≤ 0.05 was used to determine the difference between mean values. Standard deviation was provided for each mean value.

## 3 Results and discussion

### 3.1 Effect of pH on the BSA structure in aqueous solution

#### 3.1.1 Effect of pH on zeta potential of BSA

**Fig. 3:**
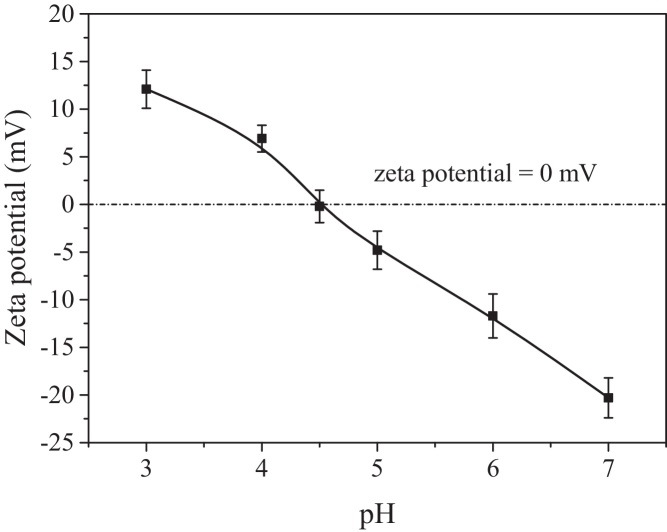
Effect of pH on zeta potential of BSA at BSA concentration of 15.0 g/L.

Zeta potential, as a characterization of electrostatic repulsion between protein molecules, has a great importance in understanding the protein adsorption at the gas–liquid interface at the molecular level [23]. Thus the effect of pH on the zeta potential of BSA was investigated in this section. The results are presented in Fig. 3. From Fig. 3, the zeta potential of BSA molecules increased from −20.3 ± 2.1 mV to −0.2 ± 1.7 mV to 12.1 ± 2.0 mV with decreasing pH from 7.0 to 4.5 to 3.0. The results indicate that pH 4.7 was the isoelectric point of BSA, the same as the one reported by Chai et al. [8]. Furthermore, the variation of zeta potential with pH was more significant in the pH range higher than pH 4.7 than that in the pH range lower than 4.7.

#### 3.1.2 Effect of pH on molecular size of BSA

**Fig. 4:**
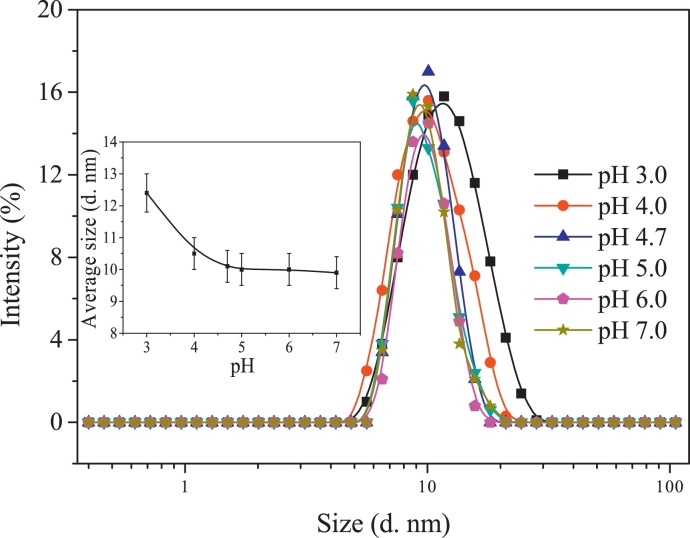
Effects of pH on molecular size distribution of BSA (main figure) and average molecular size (inserted figure) at BSA concentration of 0.30 g/L.

Molecular size is an important parameter to determine the protein adsorption at the gas–liquid interface [28], so the effect of pH on the size distribution of BSA molecules was investigated in this section. The results are presented in Fig. 4.

In the main figure of Fig. 4, BSA had the similar molecular size distribution in the pH range from 7.0 to 4.7. As pH decreased from 4.7 to 3.0, the molecular size distribution became wider. The inserted figure of Fig. 4 shows that the average molecular size suffered no significant changes with decreasing pH from 7.0 to 4.7 but largely increased from 10.1 ± 0.5 nm to 12.4 ± 0.6 nm as pH further decreased from 4.7 to 3.0. The results indicate that pH unfolded the BSA structure in the range lower than the isoelectric point of BSA. The results were similar to those of El Kadi et al. [12] and Barbosa et al. [2], where BSA had a partially unfolded structure in the pH range from 4.0 to 2.0. Combining the results in Fig. 3, Fig. 4, increasing positive charge but not negative charge unfolded the BSA structure. It is indicated that the acid amino acid residues were mainly present at the molecular surface of BSA while the basic ones were distributed inside the BSA molecule. When the BSA structure was unfolded at a low pH, more basic amino acid residues were exposed to the solution and accessible to ionization. However, in the pH range from 4.7 to 3.0, the molecular size also increased and then the increase of the electric charge density would be smaller than that in the pH range from 4.7 to 7.0.

#### 3.1.3 Effect of pH on second structure of BSA

**Fig. 5:**
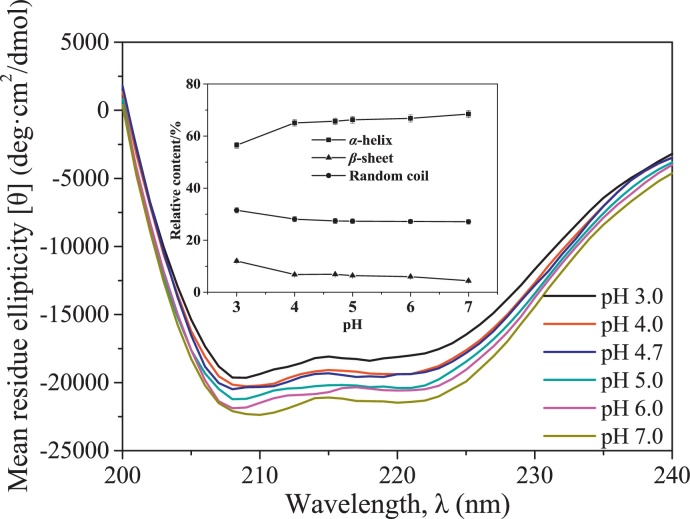
Effects of pH on mean residue ellipticity (main figure) and secondary structure compositions of BSA (inserted figure) at BSA concentration of 0.30 g/L.

BSA has an α-helix-dominant second structure, so the decrease in the relative content of α-helix often corresponds to the unfolding of the BSA structure [25]. Furthermore, Bhattacharya et al. [4] reported that increasing the relative content of β-sheet enhanced the aggregation of BSA, suggesting the importance of secondary structure in the aggregation of BSA. Thus the effect of pH on the secondary structure of BSA was investigated. The results are presented in Fig. 5.

In the main figure of Fig. 5, at a fixed wavelength from 190 nm to 240 nm, the negative value of mean residue ellipticity gradually increased with decreasing pH from 7.0 to 3.0. It is indicated that pH affected the secondary structure of BSA. In detail, the inserted figure of Fig. 5 shows that the relative contents of α-helix, β-sheet and random coil had no significant changes with decreasing pH from 7.0 to 4.0. As pH further decreased to pH 3.0, the relative content of α-helix significantly decreased with an obvious increase in the relative content of β-sheet. [25] reported that the relative content of α-helix of BSA at pH 7.0 (67%) was much higher than that at pH 2.8 (54%). Their results were basically in consistent with the current ones. For BSA, the decrease in the relative content of α-helix indicates the unfolding of the protein structure. Thus the results in Fig. 5 were also consistent with those in Fig. 4.

#### 3.1.4 Effect of pH on hydrophobicity of BSA

**Fig. 6:**
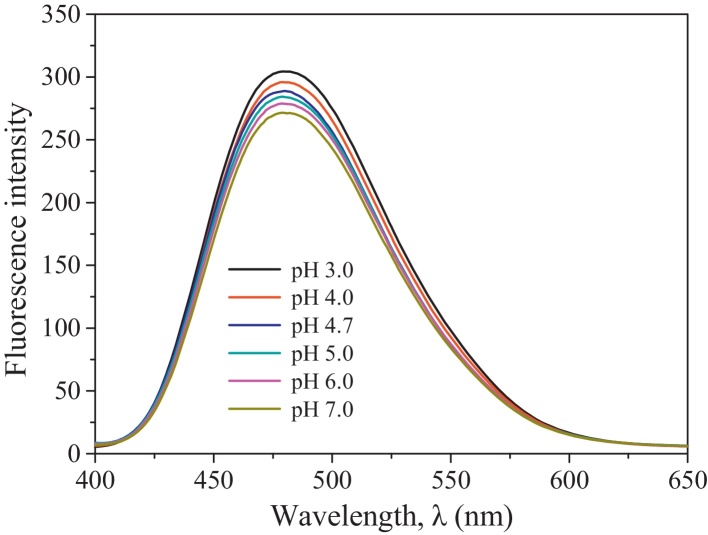
Effect of pH on extrinsic fluorescence spectrum of BSA solution at BSA concentration of 0.30 g/L.

Hydrophobicity plays an important role in the adsorption of proteins at the gas–liquid interface [27]. Thus in this section, the effect of pH on the hydrophobicity of BSA was investigated using extrinsic fluorescence spectrometry with ANS as a fluorescence probe [26]. The specific measurements for the extrinsic fluorescence spectra of BSA solutions had been described in the previous work of Li et al. [17]. The results are presented in Fig. 6. From Fig. 6, the maximal fluorescence intensity gradually increased with decreasing pH from pH 7.0 to pH 3.0. It is indicated that decreasing pH increased the BSA hydrophobicity. The decrease in pH from 7.0 to 4.7 reduced the negative charge which could prevent the ANS probe from interacting with the hydrophobic groups of the BSA molecule. As pH further decreased, the BSA structure became unfolded so that more ANS probes were associated with the hydrophobic groups. In addition, the increased positive net charge could enhance the interaction between the BSA molecules and the ANS probes [26]. So the maximal fluorescence intensity increased as pH decreased.

### 3.2 Role of pH-induced structural change in the aggregation of BSA in aqueous solution

**Fig. 7:**
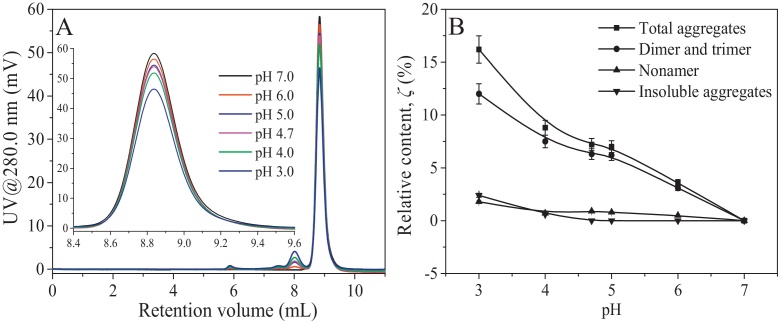
Effects of pH on SEC spectrum of BSA solution (A) and relative contents of total aggregates, dimer and trimer, nonamer and insoluble aggregates (B) at BSA concentration of 0.30 g/L. The peaks at about 8.95 mL, 8.01 mL, 7.46 mL and 5.90 mL correspond to monomer, dimer, trimer and nonamer of BSA, respectively.

For clearly understanding how pH affected the aggregation of BSA induced at the gas–liquid interface, the role of pH-induced structural change in the aggregation of BSA in aqueous solution was investigated by correlating the effect of pH on the aggregation of BSA with its effect on the BSA structure. In the experiments, the BSA sample without aggregates was prepared by size exclusion chromatography according to the work of [17]. It was used to prepare the BSA solutions of 0.30 g/L with pH 7.0, 6.0, 5.0, 4.7, 4.0 and 3.0. The prepared BSA solution at each pH was stored at 4 °C for 24 h and then the relative content of each BSA aggregate (*ζ*) in it was detected. The results for the effect of pH on the aggregation of BSA in the aqueous solution are presented in Fig. 7.

Fig. 7A shows that the peak area for the BSA monomer gradually reduced as pH decreased from pH 7.0 to 3.0, corresponding to the increase in the peak area of BSA aggregates, particularly dimer. Specifically, Fig. 7B shows that *ζ*_total aggregates_ increased from 0% to 16.2 ± 1.3% with decreasing pH from 7.0 to 3.0. More importantly, *ζ*_dimer and trimer_ significantly increased as pH decreased and insoluble aggregates were formed when pH was lower than 4.0. In addition, *ζ*_nonamer_ had no significant changes with pH. Thus in the current pH range, decreasing pH enhanced the formation of dimer and even insoluble aggregates. Referring to the results in Section 3.1, decreasing pH from 7.0 to 4.7 reduced the net charge of BSA molecules but did not change their structures. Then, the decrease in pH just weakened the intermolecular electrostatic repulsion to improve the possibility for protein aggregation. As pH decreased from 4.7 to 3.0, the BSA structure unfolded to weaken the intermolecular repulsion and make more hydrophobic groups exposed to the solution so that the BSA aggregation was also enhanced. The increased relative content of β-sheet could also intensify the aggregation of BSA [4]. Furthermore, the more unfolded structure allowed more protein molecules to form an aggregate with a larger size. Resultantly, reducing pH enhanced the aggregation of BSA and even the formation of insoluble aggregates. In addition, the unfolding of the protein structure played a more important role in protein aggregation than the weakened intermolecular repulsion.

### 3.3 Role of pH-induced structural change in the aggregation of BSA in foam fractionation

#### 3.3.1 Effect of pH on the adsorption of BSA at the gas–liquid interface

**Fig. 8:**
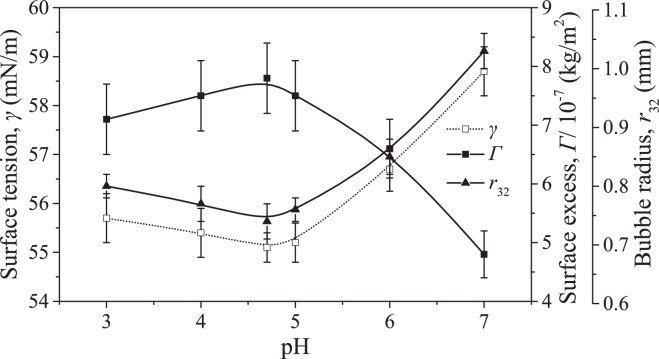
Effects of pH on surface tension of BSA solution, surface excess of BSA and bubble radius at BSA concentration of 0.30 g/L.

Before the studies on the effect of pH on the aggregation of BSA in foam fractionation, its role in the adsorption of BSA at the gas–liquid interface was firstly discussed by investigating its effects on the surface tension of the BSA solution (*γ*), and the surface excess of BSA (Γ) and bubble radius (*r*_32_) during the process of foam fractionation. The measurements of Γ and *r*_32_ had been specifically described in the previous work of Li et al. [17] with the pictures for measuring *r*_32_ taken at the top of the foam fractionation column. The results are presented in Fig. 8.

From Fig. 8, Γ slightly sharply increased from (4.8 ± 0.4) × 10^−7^ kg/m^2^ to (7.8 ± 0.6) × 10^−7^ kg/m^2^ and then slightly decreased to (7.1 ± 0.6) × 10^−7^ kg/m^2^ as pH decreased from 7.0 to 4.7 to 3.0. Correspondingly, *γ* suffered the inverse variation with pH. At the isoelectric point (pH 4.7), BSA had the maximal adsorption at the gas–liquid interface and the result was consistent with the common knowledge [13]. The sharp increase in Γ with decreasing pH from 7.0 to 4.7 was attributed to the gradually weakened intermolecular electrostatic repulsion. Importantly, the statistical analysis shows that the variation of Γ in the pH range from 4.7 to 3.0 was not significant, although the intermolecular electrostatic repulsion and the BSA molecular size increased. In general, Γ decreased as pH deviated from the protein isoelectric point due to the enhancement in the intermolecular electrostatic repulsion. Furthermore, the increase in molecular size also decreased the protein adsorption at the gas–liquid interface due to the increase in diffusion resistance [28]. However, both the two effects did not significantly reduce Γ. It is indicated that the protein–protein association at the gas–liquid interface played an important role in preventing the decrease in Γ with decreasing pH from 4.7 to 3.0. The adsorption of BSA at the gas–liquid interface obeyed the multilayer adsorption theory so that a higher level in protein–protein association at the gas–liquid interface corresponded to a higher surface excess [11]. The results in Section 3.1 show that decreasing pH from 4.7 to 3.0 made the BSA structure more unfolded to increase the protein hydrophobicity. Then the unfolded BSA molecules with high hydrophobicity certainly enhanced the protein-protein association at the gas–liquid interface [22]. As a result, Γ had no significant decrease in the pH range from 4.7 to 3.0. In addition, *r*_32_ significantly decreased to the lowest value and then slightly increased as pH decreased from 7.0 to 4.7 to 3.0. According to the Laplace equation, a lower Γ corresponded to a smaller *r*_32._ So the variation of *r*_32_ with pH was opposite to that of Γ. That is to say, larger bubbles were stabilized by a smaller amount of BSA molecules adsorbed at the gas–liquid. In this case, the adsorbed BSA molecules undoubtedly had more unfolded structures to make more hydrophobic groups exposed at the gas phase to stabilize the bubbles [7]. Resultantly, a lower Γ corresponded to a more unfolded BSA structure at the gas–liquid interface [21].

#### 3.3.2 Effect of pH on the aggregation of BSA in foam fractionation

**Fig. 9:**
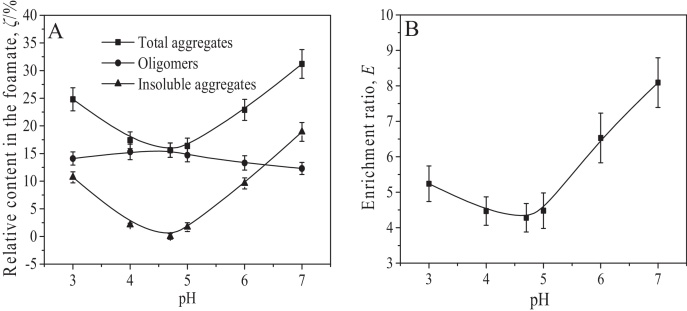
Effects of pH on the relative contents of total aggregates, oligomers (132.8–664 kDa) and insoluble aggregates in the foamate (A) and the BSA enrichment ratio (B).

In this section, the role of pH in the aggregation of BSA in foam fractionation was discussed by analyzing the effects of pH on the relative contents of BSA aggregates in the foamate (*ζ*) and its effects on the structure and interfacial adsorption of BSA. The results for the effects of pH on the relative contents of BSA aggregates in the foamate and the BSA enrichment ratio (*E*) are presented in Fig. 9.

From Fig. 9A, *ζ*_total aggregates_ decreased from 31.2 ± 22.6% to 15.6 ± 1.3% and then increased to 24.8 ± 2.1% with decreasing pH from 7.0 to 4.7 to 3.0. Furthermore, the variation of *ζ*_total aggregates_ with pH was attributed to that of *ζ*_insoluble aggregates_ with pH. At the isoelectric point, BSA suffered the lowest level in protein aggregation induced by the gas–liquid interface and no insoluble aggregates were formed in the foamate. As pH deviated from the isoelectric point, the increase in *r*_32_ resulted in a gradual decrease in the liquid holdup of the rising foam so that *E* gradually increased (Fig. 9B). A high *E* corresponded to a high ratio of the amount of BSA molecules adsorbed at the gas–liquid interface vs that in the entrained liquid. So a high *E* readily resulted in a high level protein aggregation in the defoaming process [17]. The discussion in Section 3.3.1 suggests that the structures of the BSA molecules adsorbed at the gas–liquid interface became gradually unfolded with pH deviating from the isoelectric point. The gradual unfolding protein structure also enhanced the aggregation of BSA molecules in their desorption from the gas–liquid interface. Resultantly, *ζ*_total aggregates_ had the lowest value at the isoelectric point. Specifically, in the pH range from 7.0 to 4.7, *ζ*_total aggregates_ and *E* suffered similarly sharp decreases. It is indicated that most BSA aggregates were formed in the defoaming process [17]. As pH decreased from 4.7 to 3.0, *E* slightly increased while *ζ*_total aggregates_ largely increased. The results are mainly because the gradual unfolding of the BSA structure caused a high level in protein aggregation at the gas–liquid interface and the aggregates formed at the gas–liquid interface further aggregated to insoluble ones in their desorption from the interface.

## 4 Conclusions

Decreasing pH from 7.0 to 3.0 gradually unfolded the BSA structure to increase the molecular size and the relative content of β-sheet. The unfolding structure enhanced the formation of insoluble aggregates in the aqueous solution and thus increased the instability of BSA. At the isoelectric point (pH 4.7), no remarkable experimental phenomenon on the aggregation of BSA in the aqueous solution was observed. In addition, the unfolding of the protein structure played a more important role than the zero net charge in the enhancement of protein aggregation.

At the isoelectric point, BSA suffered the lowest level in protein aggregation induced by the gas–liquid interface. As pH deviated from the isoelectric point, the increase in bubble size resulted in the increase in the BSA enrichment ratio. The increased enrichment ratio enhanced the formation of insoluble aggregates in the desorption of BSA molecules from the gas–liquid interface. In the pH range from 7.0 to 4.7, most BSA aggregates were formed in the defoaming process. In the pH range from 4.7 to 3.0, the unfolding of the BSA structure enhanced protein aggregation at the gas–liquid interface and the aggregates at the interface further aggregated to insoluble ones in the desorption process. The results had important implications to clearly understanding the role of pH in protein aggregation in foam fractionation.

## References

[1] Barackov I., Mause A., Kapoor S., Winter R., Schembecker G., Burghoff B. (2012). Investigation of structural changes of β-casein and lysozyme at the gas–liquid interface during foam fractionation. J. Biotechnol..

[2] Barbosa L.R., Ortore M.G., Spinozzi F., Mariani P., Bernstorff S., Itri R. (2010). The importance of protein-protein interactions on the pH-induced conformational changes of bovine serum albumin: a small-angle X-ray scattering study. Biophys. J..

[3] Bee J.S., Schwartz D.K., Trabelsi S., Freund E., Stevenson J.L., Carpenter J.F., Randolph T.W. (2012). Production of particles of therapeutic proteins at the air–water interface during compression/dilation cycles. Soft Matter.

[4] Bhattacharya A., Prajapati R., Chatterjee S., Mukherjee T.K. (2014). Concentration-dependent reversible self-oligomerization of serum albumins through intermolecular β-sheet formation. Langmuir.

[5] Brown L., Narsimhan G., Wankat P.C. (1990). Foam fractionation of globular proteins. Biotechnol. Bioeng..

[6] Burghoff B. (2012). Foam fractionation applications. J. Biotechnol..

[7] Clarkson J.R., Cui Z.F., Darton R.C. (1999). Protein denaturation in foam: I. Mechanism study. J. Colloid Interface Sci..

[8] Chai C., Lee J., Huang Q. (2014). The effect of ionic strength on the rheology of pH-induced bovine serum albumin/κ-carrageenan coacervates. LWT Food Sci. Technol..

[9] Chi E.Y., Krishnan S., Randolph T.W., Carpenter J.F. (2003). Physical stability of proteins in aqueous solution: mechanism and driving forces in nonnative protein aggregation. Pharm. Res..

[10] Cromwell M.E., Hilario E., Jacobson F. (2006). Protein aggregation and bioprocessing. AAPS J..

[11] Douillard R., Lefebvre J. (1990). Adsorption of proteins at the gas–liquid interface: models for concentration and pressure isotherms. J. Colloid Interface Sci..

[12] El Kadi N., Taulier N., Le Huerou J.Y., Gindre M., Urbach W., Nwigwe I., Kahn P.C., Waks M. (2006). Unfolding and refolding of bovine serum albumin at acid pH: ultrasound and structural studies. Biophys. J..

[13] Engelhardt K., Lexis M., Gochev G., Konnerth C., Miller R., Willenbacher N., Peukert W., Braunschweig B. (2013). pH effects on the molecular structure of β-lactoglobulin modified air–water interfaces and its impact on foam rheology. Langmuir.

[14] Kou Q.Y., Wu Z.L., Hu N. (2015). Thermodynamic adsorption properties of bovine serum albumin and lysozyme on the bubble surface from the binary solution. Chem. Eng. Res. Des..

[15] Lambert W.D., Du L., Ma Y., Loha V., Burapatana V., Prokop A., Tanner R.D., Pamment N.B. (2003). The effect of pH on the foam fractionation of β-glucosidase and cellulase. Bioresour. Technol..

[16] Li D., Zhu M., Xu C., Ji B. (2011). Characterization of the baicalein–bovine serum albumin complex without or with Cu^2+^ or Fe^3+^ by spectroscopic approaches. Eur. J. Med. Chem..

[17] Li R., Fu N., Wu Z., Wang Y., Wang Y. (2015). Protein aggregation in foam fractionation of bovine serum albumin: effect of protein concentration. Biochem. Eng. J..

[18] Li R., Wu Z., Wang Y., Liu W. (2014). Pilot study of recovery of whey soy proteins from soy whey wastewater using batch foam fractionation. J. Food Eng..

[19] Liu Z., Liu Z., Wang D., Ding F., Yuan N. (1998). On the denaturation of enzymes in the process of foam fractionation. Bioseparation.

[20] Lemlich R. (1968). Adsorptive bubble separation methods-foam fractionation and allied techniques. Ind. Eng. Chem. Res..

[21] Miller R., Fainerman V.B., Makievski A.V., Krägel J., Grigoriev D.O., Kazakov V.N., Sinyachenko O.V. (2000). Dynamics of protein and mixed protein/surfactant adsorption layers at the water/fluid interface. Adv. Colloid Interface Sci..

[22] Oboroceanu D., Wang L., Magner E., Auty M.A. (2014). Fibrilization of whey proteins improves foaming capacity and foam stability at low protein concentrations. J. Food Eng..

[23] Pezennec S., Gauthier F., Alonso C., Graner F., Croguennec T., Brule G., Renault A. (2000). The protein net electric charge determines the surface rheological properties of ovalbumin adsorbed at the air–water interface. Food Hydrocolloids.

[24] Rangarajan V., Sen R. (2013). An inexpensive strategy for facilitated recovery of metals and fermentation products by foam fractionation process. Colloids Surf. B.

[25] Takeda K., Wada A., Yamamoto K., Moriyama Y., Aoki K. (1989). Conformational change of bovine serum albumin by heat treatment. J. Protein Chem..

[26] Tang C.H., Shen L. (2013). Role of conformational flexibility in the emulsifying properties of bovine serum albumin. J. Agric. Food Chem..

[27] Townsend A.A., Nakai S. (1983). Relationships between hydrophobicity and foaming characteristics of food proteins. J. Food Sci..

[28] Vogler E.A. (2012). Protein adsorption in three dimensions. Biomaterials.

[29] Wang W. (2005). Protein aggregation and its inhibition in biopharmaceutics. Int. J. Pharm..

[30] Yang Q., Wu Z., Yin H., Tan Y. (2011). Enhancing foam drainage using foam fractionation column with spiral internals for separation of bovine serum albumin. CIESC J..

